# A Fiber Bragg Grating (FBG)-Enabled Smart Washer for Bolt Pre-Load Measurement: Design, Analysis, Calibration, and Experimental Validation

**DOI:** 10.3390/s18082586

**Published:** 2018-08-07

**Authors:** Dongdong Chen, Linsheng Huo, Hongnan Li, Gangbing Song

**Affiliations:** 1 State Key Laboratory of Coastal and Offshore Engineering, Dalian University of Technology, Dalian 116024, China; chendongdlut@mail.dlut.edu.cn (D.C.); hnli@dlut.edu.cn (H.L.); 2 School of Civil Engineering, Shenyang Jianzhu University, Shenyang 110168, China; 3 Smart Material and Structure Laboratory, Department of Mechanical Engineering, University of Houston, Houston, TX 77204, USA

**Keywords:** bolt pre-load measurement, bolt looseness monitoring, bolted connection monitoring, Fiber Bragg Gratings (FBG), FBG-enabled smart washer, smart washer

## Abstract

A washer is a common structural element that is directly used along the loading path of a bolted connection. Pre-load on a bolted connection directly impacts its load bearing capacity and pre-load monitoring is an important aspect of structural health monitoring (SHM). With the change of the pre-load on a bolted connection, the loading force on the washer will change and, therefore, the outer diameter and outer circumferential length of the washer will change. Taking advantage of the high sensitivity and the small size of a Fiber Bragg Grating (FBG) sensor, we propose an innovative smart washer encircled by an FBG sensor that can directly measure the circumferential strain change and, therefore, the pre-load on the washer. For protection, the FBG is embedded in a pre-machined groove along the circumferential surface of the washer. A theoretical approach is used to derive the linear relationship between the applied load and the circumferential strain of the washer. To validate the functionality of the FBG-enabled smart sensor for in situ bolt pre-load monitoring, a simple but effective testing apparatus is designed and fabricated. The apparatus involves a bolt, the FBG-enabled washer, a metal plate, and a nut. The bolt has an embedded FBG along its axial direction for precise axial strain and, therefore, force measurement. With the calibrated axial force measuring bolt, in situ experiments on the FBG-enabled smart washers are conducted. Experimental results reveal the linear relationship between the pre-load and the wavelength of the FBG sensor encircling the washer. Both analytical and experimental results demonstrate that the proposed novel approach is sensitive to the bolt pre-load and can monitor in real time the bolt looseness in the entire loading range.

## 1. Introduction

As one of the most common types of structural connection, bolted joints are commonly used in civil structures, such as long-span bridges and high-rise buildings [1,2]. The pre-load of a bolt connection directly impacts its load bearing capacity and plays an important role in the structural wellbeing [1,2]. With the recent emphasis on and development of Structural Health Monitoring (SHM) [3,4,5,6,7], bolt pre-load monitoring has begun to receive attention and various methods have been proposed [8,9,10]. Wang et al. [11] conducted a review on bolted connection monitoring, which reviewed the acoustoelastic method [12], the piezoelectric active sensing method [13], and the piezoelectric impedance method [14]. 

The principle of the acoustoelastic method is based on the velocity difference of the ultrasonic wave at different axial stresses. Yasui et al. [15] proposed a new acoustoelastic axial stress measurement method in which both longitudinal and transverse waves were excited simultaneously by a combined Longitudinal/Shear (L/S) mode transducer. However, the method requires bulky equipment for accurate measurement of the ultrasonic velocity and is not suitable for real-time monitoring. On the other hand, the piezoelectric active sensing method needs only two small piezoceramic patches, which are bonded on different parts of the bolted connection, and a relatively inexpensive data acquisition system [16]. Due to its strong piezoelectric effect, Lead Zirconate Titanate (PZT) is the most commonly used piezoceramic material. In the active sensing approach, one PZT transducer is used as an actuator to generate a stress wave, and the other transducer is used as a sensor to detect the propagated wave. The generated wave travels along the loading path and crosses the interface of the bolted connection, and the propagated stress wave will then be detected by the piezoceramic sensor. The pre-load on a bolt connection directly impacts the interface of the connection, and a larger force ensures a better connection, resulting in better wave propagation with less energy dissipation. On the other hand, a lesser force creates a poor connection, resulting in poor wave propagation with more energy dissipation. A novel PZT-based smart washer was proposed by Huo et al. [17] to enable the active sensing approach for bolted connections. The PZT-based smart washer was formed by embedding a PZT patch between two metal rings. With its sensing and actuating abilities, the washer can detect the bolt pre-load looseness level through the active sensing approach. For the PZT-based active sensing method, saturation occurred when the applied torque reached a certain high value [16,17], and the method cannot detect the looseness when the saturation occurs. The theoretical analysis performed by Huo et al. [18] also validated this saturation phenomenon.

In addition, the piezoelectric impedance method has been applied to detect damage [19,20,21,22] and was first presented by Liang et al. [23]. A real-time damage detection method based on PZT impedance was investigated in pipes connected by bolted joints [24]. Data collected from the tests illustrated the capability of this method to detect the loosening of the bolted joints. A bolted loosening experiment was conducted in the work of Park et al. [25]. In their research, the mechanical impedance sensing region was investigated in a 1/4-scale bridge section specimen. Recently, the applications of wireless monitoring systems have increased rapidly [26]. Perera et al. developed a flexible wireless smart sensor (WSS) framework based on the Electromechanical Impedance (EMI) method using active sensors for full-scale and autonomous SHM [27]. With the help of unmanned aerial vehicles (UAVs), an impedance-based nondestructive health monitoring method was proposed by Na et al. [28]. In general, the impedance method is a qualitative approach to detecting local damage. This approach cannot detect minor looseness of bolt connections. In addition, piezoceramic-based active sensing or impedance methods are not suitable for monitoring systems with strong electric or magnetic fields, such as those near transformers or electric power lines.

In the structural health monitoring field, Fiber Optical Sensors (FOSs) have been widely used [29,30], including in civil structures [31], aviation [32], oil and gas [33], nuclear power plants [34], and other fields [18,35]. A Fiber Bragg Grating (FBG) is a common fiber optical sensor and has the advantages of small size, high sensitivity, electromagnetic immunity (EMI), and suitability for embedment. Plenty of studies have referred to bolt tension monitoring using FBG sensors. A bolt tension monitoring device was proposed by Khomenko et al. [36], and it utilizes removable and reusable fiber Bragg grating sensor(s) embedded in a bolt shaft for pre-load and retained clamping force measurements. They found that, depending on the joint configuration, the pre-load reduction varied considerably, and an increase in initial pre-load tended to reduce the pre-load relaxation [37]. Although this device can directly detect the defects of bolt joints, a drilled hole along the axis of the bolt through one-half of its length was required, and the drilled holed may reduce the bearing capacity of the bolt. Yeager et al. [38] presented an embedded fiber Bragg grating sensor by using the full width at half-maximum bandwidth of the Bragg reflection spectrum for monitoring the pre-load torque in a composite bolted connection. 

This paper proposes a new approach to quantitative bolt looseness monitoring by using a Fiber Bragg Grating enabled smart washer for the entire range of the applied torque. In this research, the FBG sensor was embedded into a pre-machined circumferential groove which was located in the exterior washer surface. Since the washer is directly on the loading path of a bolted connection, the FBG-enabled smart washer is very sensitive to the pre-load changes and it can continue to monitor the pre-load during the entire loading range. By analyzing the wavelength change, different bolt looseness statuses can be monitored quantitatively. Experimental results demonstrate that the FBG-enabled smart washer is a simple, feasible, and quantitative method to monitor the pre-load level. 

## 2. Fiber Bragg Grating (FBG)-Enabled Smart Washer: Principle and Design

**Figure 1:**
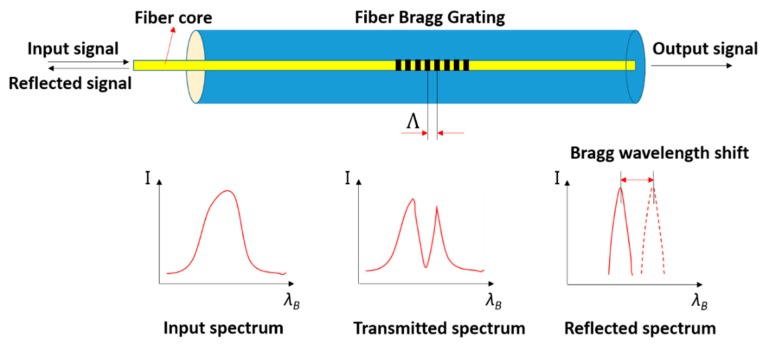
Schematic diagram of a fiber Bragg grating (FBG) sensor.

The fiber Bragg grating, as shown in Figure 1, is a wavelength-dependent filter/reflector formed by introducing a periodic refractive index structure, with spacing on the order of a wavelength of light, within the core of an optical fiber [39]. When a light passes through the grating at a particular wavelength, called the Bragg wavelength, the light will be reflected [40]. The Bragg wavelength is expressed as

(1) $$\lambda_{B}=2n\Lambda$$

where *λ_B_* is the Bragg wavelength, *n* is the effective refractive index of the FBG, and *Λ* is the grating period. 

When a broad-spectrum light signal is input to the Fiber Bragg Grating section, a reflected spectrum whose center wavelength is *λ_B_* will be reflected and the remaining portion of light will transmit through. The reflected light can be used as an index to measure strain, temperature, or polarization changes by the Bragg wavelength shift. In this study, a commercially available coated FBG sensor was used to enable a washer to have the function to monitor the pre-load of a bolted connection. The length of the Bragg grating is 10 mm, the outer diameter of the fiber is 250 μm, and the diameter of the optical fiber core is 9 μm. 

**Figure 2:**
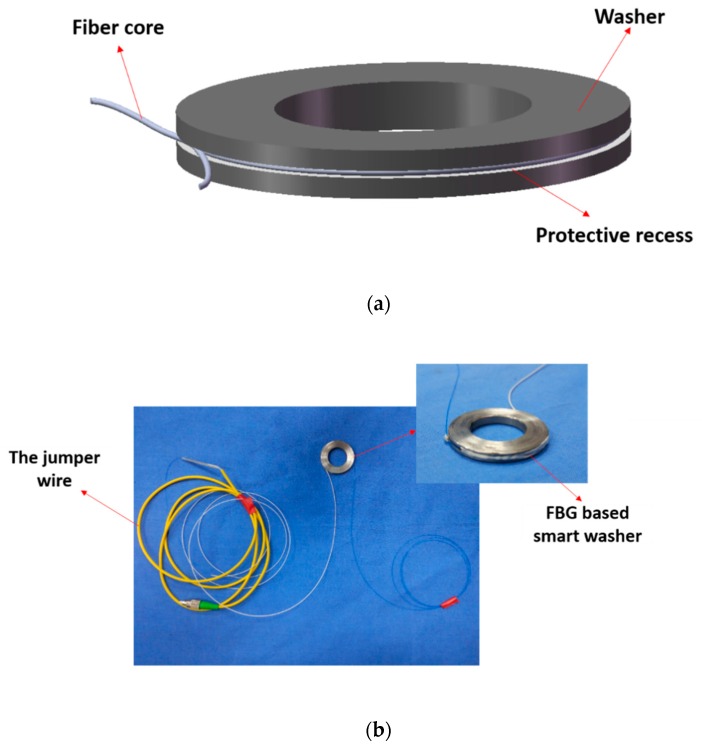
The FBG-enabled smart washer sensor. (**a**) The design diagram of the FBG-enabled smart washer; (**b**) Photos of the FBG-enabled smart washer.

The smart washer design is illustrated in Figure 2a. The smart washer is formed by encircling the washer by an optical fiber with an FBG sensor. First of all, a smooth groove was pre-machined along the outer surface of the washer. Then, 502 glue was used to bond the fiber to the washer, after the sensor was attached to the washer. Epoxy resin was then used to strengthen the strain transfer between fiber and washer. Last, 704 glue was used to protect the fiber from damage. Figure 2b shows photos of a fabricated smart washer. In this research, the inner and outer diameters of the smart washer are 23 mm and 37 mm, respectively. The height is 5 mm. The depth and width of the groove are 1.5 mm and 2 mm, respectively. With the change of the pre-load on a bolted connection, the loading force on the washer will change and, therefore, the outer diameter and outer circumferential length of the washer will change, which is directly measured by the FBG sensor. Taking advantage of the FBG sensor, including its high sensitivity, the proposed smart washer can accurately measure the pre-load on a bolted joint and detect the looseness of a bolted joint, as demonstrated in later sections.

For the smart washer with the FBG sensor, the delicate part is the fiber with gratings (about 10 mm), which is embedded in the pre-machined groove on the washer with protection of glue and epoxy. The rest of the fiber can use commercially available steel-reinforced or Kevlar-reinforced fiber. Therefore, the smart washer design is suitable for practical applications.

## 3. Smart Washer—An Analytical Model

**Figure 3:**
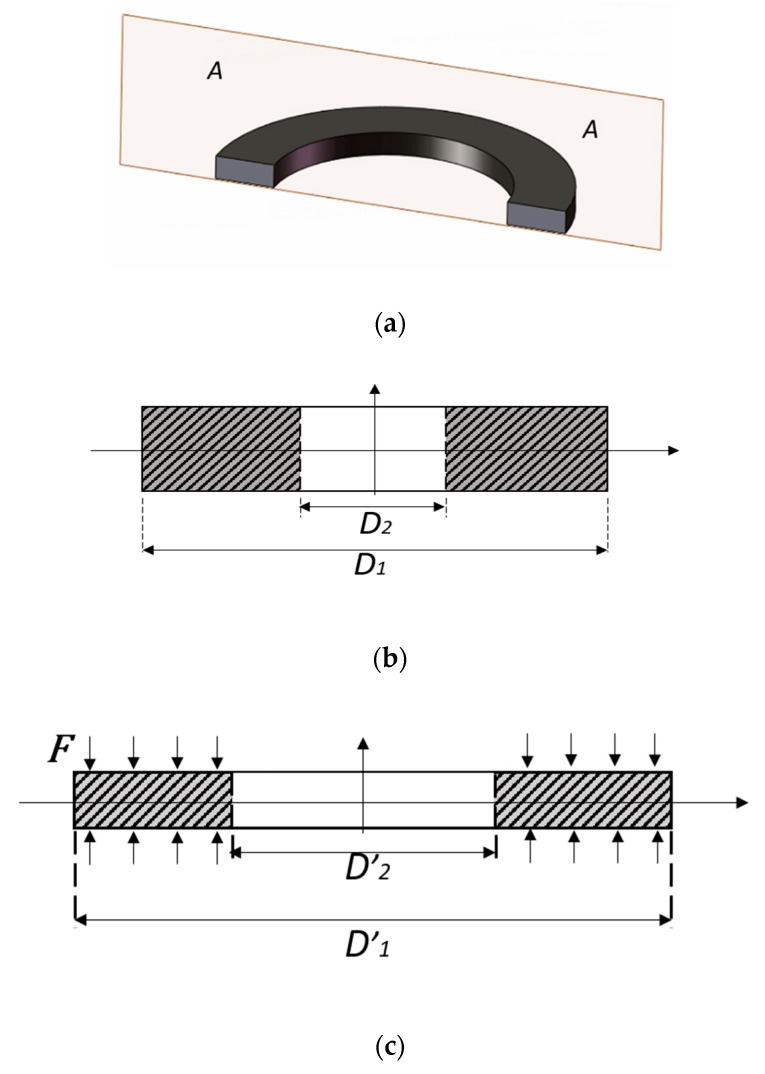
Figures to illustrate that pre-load changes induce circumferential strain change of a washer. (**a**) A 3D view of a washer with A-A cross-sectional view; (**b**) A 2D A-A cross-sectional view of washer without torque applied; (**c**) The 2D A-A cross-sectional view of washer with torque applied.

The smart washer that is proposed to monitor the bolt pre-load looseness is based on monitoring of the circumferential strain change by an FBG sensor whose wavelength changes with the circumferential strain. It is necessary to analyze the relationship between the pre-load and the circumferential strain. An in-service washer model is shown in Figure 3, which clearly shows that pre-load on the washer induces circumferential strain changes for the washer. 

An axial force on the bolt results in a uniform stress on the washer upper and lower surfaces, and the stress change can be expressed as

(2) $$\Delta \sigma =\frac{F}{A}$$

where Δ*σ* is the axial stress change of the washer, and *A* is the washer’s upper surface area which is given as

(3) $$A=\frac{\pi ({\left(D_{1}\right)}^{2}-{\left(D_{2}\right)}^{2})}{4}$$

where *D*_1_ and *D*_2_ are the external and internal nominal diameters of the washer without torque applied, and *D’*_1_ and *D’*_2_ are the external and internal nominal diameters of the washer with torque applied as shown in Figure 3b,c. The axial strain change which is represented as Δ*ε_l_* is introduced as

(4) $$\Delta \varepsilon_{l}=\frac{\Delta \sigma }{E}$$

where *E* is the Young’s modulus of the washer. According to the Poisson ratio, the transverse strain change Δ*ε_D_* can be expressed as

(5) $$\Delta \varepsilon_{D}=\mu \Delta \varepsilon_{l}.$$

Please note that the Δ*ε_D_* can also be expressed as

(6) $$\Delta \varepsilon_{D}=\frac{\Delta D_{1}}{D_{1}}=\frac{D_{1}{}^{\prime }-D_{1}}{D_{1}}$$

where Δ*D*_1_ is the pre-load decrease caused the external nominal diameter change, and the circumferential strain Δ*ε_c_* is given as

(7) $$\Delta \varepsilon_{C}==\frac{\Delta C_{1}}{C_{1}}=\frac{2\pi D_{1}{}^{\prime }-2\pi D_{1}}{2\pi D_{1}}=\frac{\Delta D_{1}}{D_{1}}$$

where *C*_1_ is the external circumferential without torque applied, and Δ*C*_1_ is the pre-load decrease caused by the external perimeter change. 

The relationship between the embedded Bragg wavelength change Δ*λ_w_* and the washer strain change Δ*ε_c_* can be presented as

(8) $$\Delta \lambda_{\omega }=k_{w}\Delta \varepsilon_{c}$$

where *k_w_* is the strain sensitivity coefficient. From Equations (6) and (7), we know that Δ*ε_c_* is equal to Δ*ε_D_*. Substituting Equations (1)–(6) into Equation (7) gives the expression of Δ*ε_c_* as

(9) $$\Delta \varepsilon_{C}=\Delta \varepsilon_{D}=\frac{\Delta D_{1}}{D_{1}}=\mu \Delta \varepsilon_{l}=\mu \frac{\Delta \sigma }{E}=\mu \frac{F}{AE}=\mu \frac{4F}{\pi E({\left(D_{1}\right)}^{2}-{\left(D_{2}\right)}^{2})}.$$

There is a linear relationship between the torque and the washer circumferential strain. The FBG wavelength can be expressed as

(10) $$\lambda_{c}=k_{w}\Delta \varepsilon_{c}+\lambda_{i}=k_{w}\mu \frac{4F}{\pi E({\left(D_{1}\right)}^{2}-{\left(D_{2}\right)}^{2})}+\lambda_{i}$$

where *λ_i_* is the initial wavelength, and *μ* and *E* are, respectively, the Poisson’s ratio and Young’s modulus of the washer.

## 4. Smart Washer—Calibration

**Figure 4:**
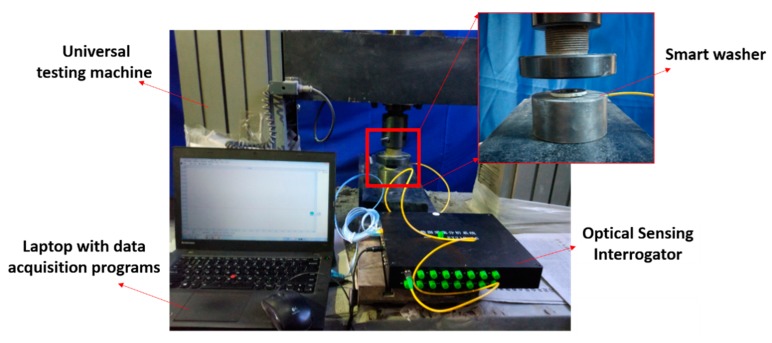
Calibration test of the FBG smart washer.

The relationship between the axial stress and the wavelength of the FBG sensor was investigated though calibration tests. Figure 4 shows the calibration setup of the calibration test. 

**Figure 5:**
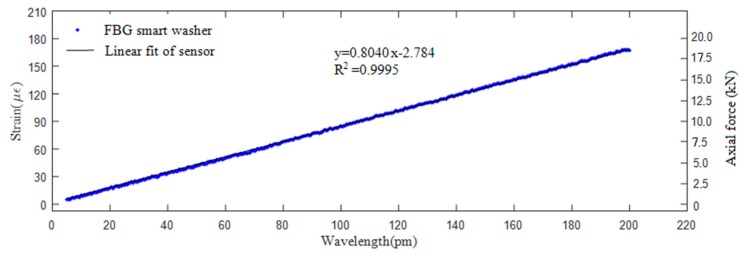
Calibration results of the smart washer.

The smart washer was loaded continuously from 0 kN to 20 kN with a load speed of 200 N/s. The strain change of the washer can be recorded by the universal testing machine. Three calibration tests were implemented and the results are plotted in Figure 5. Please note that the values of both the strain (the left ordinate) and the axial force (the right ordinate) are shown in Figure 5. The average experimental strain sensitivity coefficient of the FBG sensor is 0.8040 με/pm. The coefficient of regression association can be calculated by following equation and is found to be 0.9995:

(11) $$R^{2}=\frac{\sum_{i=1}^{n}{\left({\hat{y}}_{i}-\bar{y}\right)}^{2}}{\sum_{i=1}^{n}{\left(y_{i}-\bar{y}\right)}^{2}}$$

where ${\hat{y}}_{i}$ and *y_i_* are the calculated regression value and measured value at the *i*th point, and $\bar{y}$ is the mean value of all measured sample points. 

It is shown that the FBG smart washer sensor is stable and capable of measuring the axial stress level, which demonstrates the potential of the smart washer to monitor the looseness of a bolted connection. In addition, we did not experience noticeable loss of the light intensity for the washer that we have used during the experiments. We also noticed that there is no saturation in Figure 5, which shows the advantage of the FBG-based smart washer over the piezoceramic-based active sensing method which has the saturation issue [15,16,17]. 

## 5. Bolt Looseness Monitoring Experiments Using Smart Washer

### 5.1. Experimental Setup

**Figure 6:**
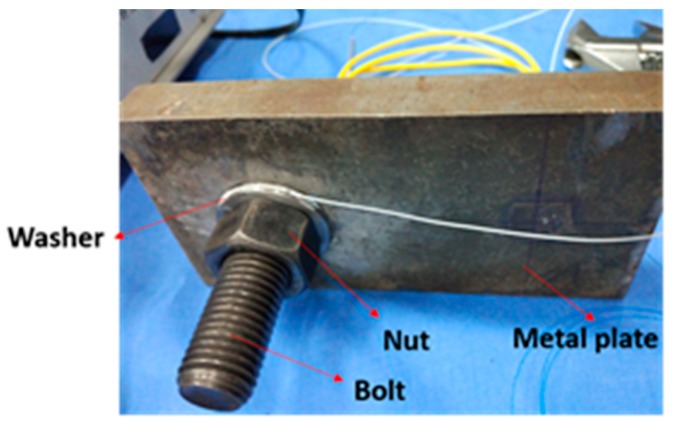
Calibration test of the FBG smart washer.

**Figure 7:**
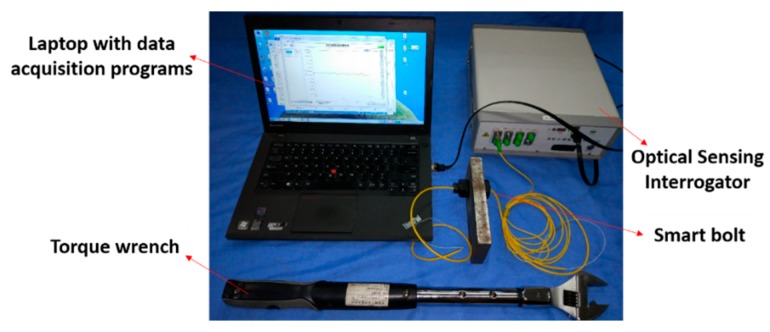
Experimental setup of external torque and bolt tension relationship verification.

As shown in Figure 6, the bolt looseness test specimen, which consists of one metal plate, a bolt, and a nut with an option of adding a washer, is used in the research. The pre-load on the bolt is controlled by a torque wrench and the stress on the bolt is measured by a smart bolt that has an embedded FBG sensor. The experimental setup is illustrated in Figure 7. The torque wrench is used to apply the required torque to the specimen to explore the relationship between the applied torque and the bolt axial force that is measured by the smart bolt. 

**Figure 8:**
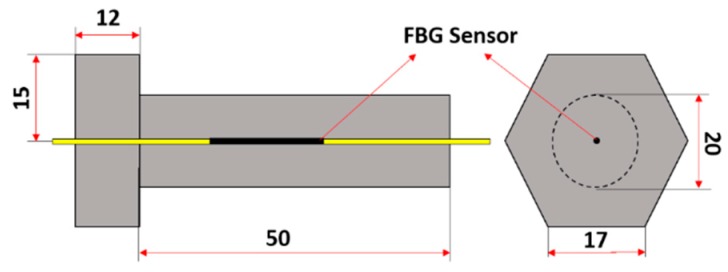
The design of a smart bolt with an embedded FBG sensor.

**Figure 9:**
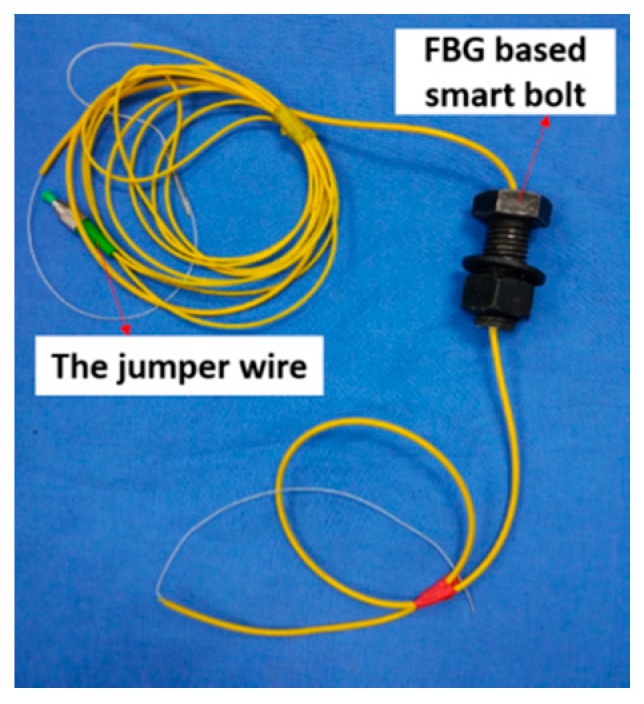
A photo of the FBG-based smart bolt that is used to measure the axial loading.

The smart bolt was made by embedding a specially treated FBG sensor which includes two gripper tubes and two mounting supports into the center of the bolt. More detailed information can be found in [41]. With the help of the FBG sensor, the smart bolt can measure the axial forces when an external torque is applied. The design and a photo of the smart bolt are shown in Figure 8 and Figure 9, respectively. 

### 5.2. Relationship Between Pre-Load and External Torque with Help from a Smart Bolt 

In the monitoring process, the bolted connection with a smart washer was fastened by applying external torque. Therefore, the relationship between applied torque and the axial load of the bolt should be first studied. The mechanical behavior of bolted joints during loosening and fastening was investigated [42,43,44,45]. The research results show that the torque applied to a bolted connection consists of three components [43,46]: (1) the torque to stretch the bolt; (2) the torque to overcome the friction in the threads of the bolt; and (3) the friction between nut face and bearing surface. In practice, it is appropriate to use the following equation to determine the torque to achieve a certain pre-load [47]:

(12) $$F=\frac{T}{k\cdot d}$$

where *F* is the axial pre-load of the bolt, *T* is the applied torque, *k* is the torque–axial load coefficient determined by factors such as bolt type and lubricant, and *d* is the nominal diameter of the bolt. 

The relationship between the smart bolt strain change Δ*ε_B_* and the embedded Bragg wavelength change Δ*λ_B_* can be presented as

(13) $$\Delta \varepsilon_{B}=k_{b}\Delta \lambda_{B}$$

where *k_b_* is the strain transfer constant coefficients. According to Equation (13), the Bragg wavelength change linearly corresponds to the smart bolt strain change. The bolt axial stress change Δ*σ_B_* is also linear with the strain change Δ*ε_B_* as

(14) $$\Delta \varepsilon_{B}=\frac{\Delta \sigma_{B}}{E_{B}}$$

where the *E_B_* is the Young’s modulus of the bolt. The bolt axial stress change Δ*σ_B_* can be expressed as

(15) $$\Delta \sigma_{B}=\frac{F}{\pi {\left(d/2\right)}^{2}}.$$

Substituting Equations (12)–(14) into Equation (15) gives the relationship between the applied torque *T* and the Bragg wavelength change Δ*λ_B_* as

(16) $$\Delta \lambda_{B}=\frac{T}{E_{B}k_{b}k\pi d{\left(d/2\right)}^{2}}.$$

To study the relationship between the axial force and the applied torque, an experiment using the FBG smart bolt [39] to monitor the axial force was conducted. Because of the limitations of the torque wrench, it can only measure the increased torque. Therefore, the reversed process of pre-load looseness was investigated this study.

**Figure 10:**
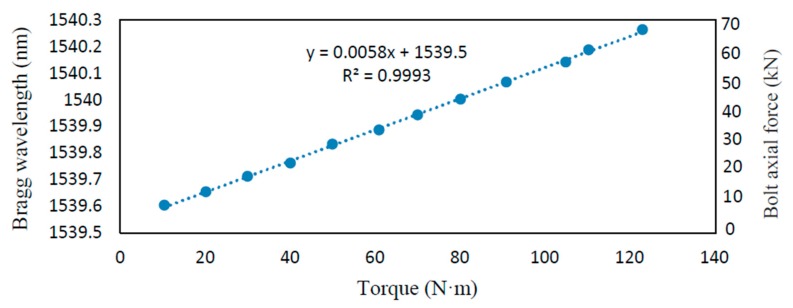
Experimental results of the linear relationship between the external torque and the Bragg wavelength changes.

The designed torque loading process is from 0–120 N·m at intervals of 10 N·m. Since the torque in this paper was applied by a manual torque wrench, there was variation in torque increment in each loading case. In order to specifically show the relationship between the external torque and wavelength change, Figure 10 is plotted and a perfect linear fitting line between the external torque and wavelength change is obtained. Please note that the values of both Bragg wavelength (the left ordinate) and the axial force (the right ordinate) are shown in Figure 5. The linear relationship validates the correctness of the analysis of Equation (16), which confirms that the FBG-based smart bolt is an accurate tool to measure the axial loading on a bolted connection.

### 5.3. Quantitative Monitoring of Bolt Pre-Load Using an FBG-Based Smart Washer

In this experiment, the pre-load is also controlled by the torque wrench. The instrumentation of the quantitative monitoring of the bolt pre-load is the same as that shown in Figure 6 and Figure 7.

**Figure 11:**
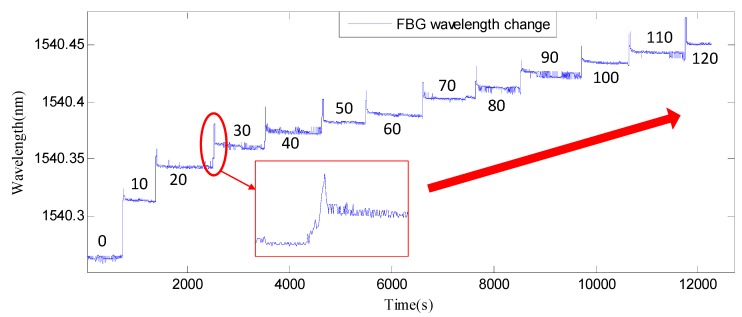
The Bragg wavelength change of the smart washer with the increased torque (Unit: N·m).

The bolt pre-load loading process is from 0–120 N·m at intervals of 10 N·m. With the increase of applied torque, the Bragg wavelength changes, as shown in Figure 11.

**Figure 12:**
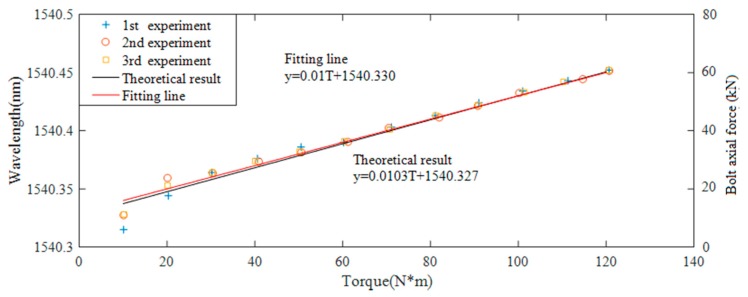
The comparison of theoretical and experimental results.

As shown in Figure 11, each step of the wavelength change is caused by the increased torque with an interval of 10 N·m from 0–120 N·m. Based on Figure 11, the relationship between the FBG wavelength and the axial force (pre-load) versus the applied torque based on experimental data is shown in Figure 12. Please note that the values of both the FBG wavelength (the left ordinate) and the axial force (the right ordinate) are shown in Figure 12. It is clear that a linear relationship is achieved without saturation. 

## 6. Validation between Theoretical and Experimental Results

In order to validate the analytical model, a comparison study of theoretical and experimental results was conducted. Substituting Equation (12) into Equation (10), the relationship between torque and the FBG smart washer wavelength can be expressed as

(17) $$\lambda_{c}=k_{w}\cdot \Delta \varepsilon_{c}+\lambda_{i}=k_{w}\cdot \mu \cdot \frac{4*T}{\pi \cdot E\cdot k\cdot d\cdot ({\left(D_{1}\right)}^{2}-{\left(D_{2}\right)}^{2})}+\lambda_{i}.$$

In this experiment, *k_w_* is 0.8040, which can be obtained from the calibration test; *μ* and *E* are the Poisson’s ratio and Young’s modulus of the washer, which are 0.32 and 2 × 10^5^ Mpa, respectively; *k* is 0.1; and *d* is the nominal diameter of the bolt, which is 0.02 m in this experiment. In addition, *D*_1_ and *D*_2_ are 0.037 m and 0.023 m, respectively. The comparison of the theoretical and three experimental results is plotted in Figure 12.

In Figure 12, it can be seen that when the torque is larger than 30 N·m, the increase of the wavelength is around 10 pm when the torque has a 10 N·m increase. The results show that it is feasible to detect the pre-load degradation by monitoring circumferential strain change of the fabricated smart washer with good repeatability. The linear relationship was obtained based on the repeated experimental data when the applied torque is higher than 30 N·m. The comparison shows that the theoretical analysis is slightly close to the experimental results. The experimental results validate the analytical ones. Once again, there is no saturation in Figure 12, which clearly shows the advantage of the FBG-based smart washer over the piezoceramic-based active sensing method which has the saturation issue [15,16,17].

## 7. Conclusions and Future Work

This paper developed a simple but effective Fiber Bragg Grating (FBG)-enabled smart washer to monitor the pre-load of a bolt connection and to detect bolt looseness during its entire loading range. The smart washer was formed by encircling the washer by an optical fiber with an FBG sensor that was embedded in a pre-machined groove along the outer surface of the washer. A theoretical approach was used to derive the linear relationship between the applied load and the circumferential strain of the washer, which was directly measured by the FBG sensor. Taking advantage of an FBG sensor including its high sensitivity, the proposed smart washer can accurately measure the pre-load on a bolted joint. To validate the functionality of the FBG-enabled smart sensor for in situ bolt pre-load monitoring, a simple but effective testing apparatus was designed and fabricated. Experimental results demonstrate the linear relationship between the pre-load and the wavelength of the FBG sensor encircling the washer. Both analytical and experimental results reveal that the proposed novel approach is sensitive to the bolt pre-load and can monitor in real time the bolt looseness in the torque loading range. Future work will include incorporation of a temperature compensation scheme with the smart washer and analytical study of strain transfer from the washer to the FBG sensor.
